# Oxygen levels affect oviduct epithelium functions in air–liquid interface culture

**DOI:** 10.1007/s00418-024-02273-1

**Published:** 2024-03-26

**Authors:** Jianchao Huo, Aleksandra Maria Mówińska, Ali Necmi Eren, Jennifer Schoen, Shuai Chen

**Affiliations:** 1 Department of Reproduction Biology, Leibniz-Institute for Zoo and Wildlife Research (IZW), Alfred-Kowalke-Straße 17, 10315 Berlin, Germany; 2 Institute of Reproductive Biology, Research Institute for Farm Animal Biology (FBN), 18196 Dummerstorf, Germany; 3 Institute of Biotechnology, Technische Universität Berlin, 13355 Berlin, Germany

**Keywords:** Oxygen, Cell culture, Air–liquid interface, Oviduct epithelium, Pig

## Abstract

Key reproductive events such as fertilization and early embryonic development occur in the lumen of the oviduct. Since investigating these processes in vivo is both technically challenging and ethically sensitive, cell culture models have been established to reproduce the oviductal microenvironment. Compartmentalized culture systems, particularly air–liquid interface cultures (ALI; cells access the culture medium only from the basolateral cell side), result in highly differentiated oviduct epithelial cell cultures. The oxygen (O_2_) tension within the oviduct is 4–10% across species, and its reduced O_2_ content is presumed to be important for early reproductive processes. However, cell culture models of the oviduct are typically cultivated without O_2_ regulation and therefore at about 18% O_2_. To investigate the impact of O_2_ levels on oviduct epithelium functions in vitro, we cultured porcine oviduct epithelial cells (POEC) at the ALI using both physiological (5%) and supraphysiological (18%) O_2_ levels and two different media regimes. Epithelium architecture, barrier function, secretion of oviduct fluid surrogate (OFS), and marker gene expression were comparatively assessed. Under all culture conditions, ALI-POEC formed polarized, ciliated monolayers with appropriate barrier function. Exposure to 18% O_2_ accelerated epithelial differentiation and significantly increased the apical OFS volume and total protein content. Expression of oviduct genes and the abundance of OVGP1 (oviduct-specific glycoprotein 1) in the OFS were influenced by both O_2_ tension and medium choice. In conclusion, oviduct epithelial cells can adapt to a supraphysiological O_2_ environment. This adaptation, however, may alter their capability to replicate in vivo tissue characteristics.

## Introduction

The luminal lining of the oviduct, comprising both ciliated and secretory epithelial cells, plays a crucial role in creating a functional environment for gamete storage and maturation, fertilization and the initial stage of embryonic development (Leese 1988; Coy et al. 2012; Li and Winuthayanon 2017; Kölle et al. 2020). The epithelium not only provides and regulates the fine-tuned dynamic availability of signalling molecules (e.g. hormones and growth factors) and nutrients within the oviduct lumen but is also involved in regulating oxygen (O_2_) tension and pH balance (Leese et al. 2001; Buhi 2002; Ng et al. 2018).

The O_2_ concentration in ambient air is approximately 21%, generally higher than in mammalian tissues. The specific localization of a tissue within the body, particularly its proximity to the O_2_-supplying capillaries (Wenger et al. 2015), determines the tissue-specific distribution of O_2_. Fertilization and early embryonic development in vivo occur in the lumen of the oviduct, an environment in which the O_2_ tension has been reported to range between 4% and 10% in various mammalian species (Fischer and Bavister 1993; García-Martínez et al. 2018; Keeley and Mann 2019). This fact has already been adopted for in vitro embryo production in humans as well as different mammalian species, demonstrating that culturing embryos under reduced O_2_ conditions better supports physiological embryonic development and leads to increased blastocyst rates, improved blastocyst quality and better pregnancy outcomes compared to ambient O_2_ level (Booth et al. 2005; Waldenström et al. 2009; Ealy et al. 2019). In addition to its role in embryo development, the O_2_ tension within the oviduct is also known to significantly influence sperm motility and competence of matured oocytes (Nevo 1965; Haidri et al. 1971).

As early reproductive events are notoriously challenging to assess in vivo, cell culture models are utilized to deepen our understanding of oviduct functions and early embryo-maternal interactions and improve assisted reproductive technologies. These in vitro studies typically occur in controlled cell culture incubators with regulated humidity and temperature. The pH of the cell culture medium is held in the physiological range of 7.2–7.4 by a bicarbonate-based buffer system and/or by adding synthetic buffers like HEPES. In standard cell culture procedures, the O_2_ level in the incubator is usually unregulated and relies on the O_2_ content of the surrounding ambient air. Depending on the height above sea level, humidified incubators running at 5% CO_2_ typically have an O_2_ level of around 18% (Wenger et al. 2015). Throughout the literature, this condition is considered as ‘normoxia’ in most experiments (Abbas et al. 2021), which, however, is hyperoxic compared to what oviductal cells encounter in vivo. The O_2_ concentration reaching cells within cell culture devices largely depends on the composition of the medium (O_2_ solubility) and the distance between cells and the medium surface. Recently, there has been a growing recognition that the supraphysiological O_2_ environment in routine cell cultures impacts cell growth, the metabolism of reactive oxygen species and gene expression profiles, leading to deviations from in vivo tissue physiology (Stuart et al. 2018; Fonseca et al. 2018; Alva et al. 2022a).

Compartmentalized in vitro systems have provided a faithful representation of the epithelial lining of the oviduct (Miessen et al. 2011; Chen et al. 2013b), allowing hormonal simulation of estrous cycle stages (Chen et al. 2013a, 2018) as well as embryo co-cultures (Chen et al. 2017). Cells are grown on porous membranes that mimic in vivo-like nutrition supply from the basolateral side of cells. After a submerged proliferation phase, the apical medium is suctioned off from the confluent epithelium, and the culture undergoes differentiation at the air–liquid interface (ALI), forming a ciliated and polarized monolayer. Proliferation and differentiation phases can be carried out using a single medium (one-step approach) or two media (two-step approach), each designed to support a particular phase (Chen and Schoen 2021). In compartmentalized culture, especially when employing the ALI approach, cells are directly exposed to the incubator atmosphere. Hence, they might be more vulnerable to the incubator’s gas composition than the conventional 2D adherent submerged cultures. Previous studies have shown that an in vivo-like morphology of oviduct epithelium in compartmentalized culture models can be achieved using various culture media regimes under varying O_2_ tensions (summarized in Table 1). However, until now, no direct comparison has been performed to assess the impact of the media regime and O_2_ level on the phenotypic and molecular features of the oviduct epithelium in a compartmentalized culture system.

**Table 1:** List of compartmentalized cultures of oviduct epithelial cells from different species. All cultures applied the air–liquid interface (ALI) technique, with the exception of the study by Ferraz et al. 2018, which utilized the liquid–liquid interface

Species	Year	Culture conditions		Media regime	Citations	Species	Year	Culture conditions		Media regime	Citations
		Temperature	O_2_					Temperature	O_2_		
*Homo sapiens*	2010	37 °C	Atmospheric	One-step	(Levanon et al. 2010)	*Sus scrofa*	2013	38 °C	Atmospheric	One-step	(Chen et al. 2013b)
*Homo sapiens*	2011	37 °C	Atmospheric	One-step	(Fotheringham et al. 2011)	*Sus scrofa*	2017	37 °C	Atmospheric	One-step	(Palma-Vera et al. 2017)
*Homo sapiens*	2020	37 °C	Atmospheric	One-step	(Brand et al. 2020)	*Sus scrofa*	2018	37 °C	Atmospheric	One-step	(Chen et al. 2018)
*Homo sapiens*	2020	–	–	One-step	(McQueen et al. 2020)	*Sus scrofa*	2019	37 °C	Atmospheric	One-step	(Zhu et al. 2019)
*Bos taurus*	2012	39 °C	Atmospheric	Two-step	(Gualtieri et al. 2012)	*Sus scrofa*	2020	37 °C	5%	Two-step	(Du et al. 2020)
*Bos taurus*	2013	39 °C	Atmospheric	Two-step	(Gualtieri et al. 2013)	*Sus scrofa*	2020	37 °C	Atmospheric	Two-step	(Zhu et al. 2020)
*Bos taurus*	2014	37 °C	Atmospheric	One-step	(Palma-Vera et al. 2014)	*Sus scrofa*	2021	37 °C	Atmospheric	Two-step	(Zhu et al. 2021)
*Bos taurus*	2016	39 °C	Atmospheric	One-step	(Simintiras et al. 2016)	*Sus scrofa*	2022	37 °C	5%	Two-step	(Du et al. 2022)
*Bos taurus*	2017	37 °C	Atmospheric	Two-step	(van der Weijden et al. 2017)	*Sus scrofa*	2023	37 °C	Atmospheric	One-step	(Zhu et al. 2023)
*Bos taurus*	2017	39 °C	Atmospheric	One-step	(Simintiras and Sturmey 2017)	*Felis catus*	2022	38.5 °C	Atmospheric	Two-step	(Eder et al. 2022)
*Bos taurus*	2017	38.5 °C	Atmospheric	One-step	(Ferraz et al. 2017)	*Equus caballus*	2022	38 °C	Atmospheric	One-step	(Leemans et al. 2022)
*Bos taurus*	2018	38.5 °C	7%	One-step	(Ferraz et al. 2018)	*Canis lupus*	2020	38.5 °C	Atmospheric	One-step	(de Almeida Monteiro Melo Ferraz et al. 2020)
*Sus scrofa*	2011	37 °C	Atmospheric	One-step	(Miessen et al. 2011)	*Monkey* (unspecified)	2006	–	–	Two-step	(Rajagopal et al. 2006)
*Sus scrofa*	2013	38 °C	Atmospheric	One-step	(Chen et al. 2013a)	*Bos Taurus,* *Sus scrofa,* *Mus musculus*	2017	37 °C	Atmospheric	Two-step	(Chen et al. 2017)

Atmospheric O_2_: approximately 18% O_2_ in humidified incubator with 5% CO_2_ at sea level

Based on a well-established ALI culture model of porcine oviduct epithelial cells (ALI-POEC), the present study aimed at comparing the influence of physiological (5% O_2_) and standard cell culture (18% O_2_) conditions on oviduct epithelial cells under two previously published media regimes, namely the one-step and two-step approaches. The assessment focused on epithelial architecture, cell barrier formation, oviduct fluid surrogate (OFS) secretion and expression of genes related to oviduct functionality.

## Materials and methods

### Media and reagents

Unless otherwise indicated, all cell culture media and supplements were purchased from Biochrom AG, Germany (part of the Merck Millipore, USA).

### Tissue collection and ALI-POEC culture

We collected oviduct tissues from 11 healthy, non-cycling 6-month-old gilts. As the tissues are by-products from a local commercial slaughterhouse (Danish Crown Teterower Fleisch GmbH, Teterow, Germany), ethical approval does not apply to this study. Within 15 min after slaughter, oviducts were excised from the connecting tissues, rinsed twice in cold Dulbecco’s phosphate buffered saline (DPBS) supplemented with 0.05 mg/ml gentamycin, 1 μg/ml amphotericin B, 100 U/ml penicillin, 100 μg/ml streptomycin and immediately kept on ice. In parallel, ovarian morphology was assessed. Oviducts were included in the study if only small follicles and no corpora lutea were visible on the respective ovaries, indicative of a lack of cyclic activity. Samples were transported on ice within 45–60 min to the laboratory for further processing. Oviduct tubes from five animals were segregated into ampullary and isthmic regions to assess histological properties (see Sect. “Histology and histomorphometry”). Oviducts from another five animals were utilized to isolate primary oviductal epithelial cells following our previously established protocols (Chen et al. 2013b; Miessen et al. 2011), involving a sequential enzymatic digestion using 1 mg/ml collagenase from clostridium histolyticum 1A (C2674, Sigma-Aldrich, USA) and Accutase (A1110501, ThermoFisher Scientific, USA) (Chen and Schoen 2021). The freshly isolated POEC were cryopreserved in Gibco Recovery Cell Culture Freezing Medium (12648010, ThermoFisher Scientific, USA) and later thawed according to the manufacturer’s instructions. Freshly isolated POEC from one animal were lysed with ice-cold RIPA buffer (9806, Cell Signaling Technology, USA), and the supernatant was collected after centrifuging at 14,000 × *g* for 30 min at 4 °C. The cell lysate served as a reference sample for western blot (see Sect. “Western blot”).

Cells acquired from each animal were placed onto 24-well inserts (83.3932.040, Sarstedt, Germany) that had been pre-coated with human collagen IV (C5533, Sigma-Aldrich, USA) at a quantity of 12 inserts per animal. The seeding density was adjusted to 1.5 × 10^5^ cells per insert. The basal compartment was filled with 1 ml of culture medium, while the apical compartment received 0.2 ml of the same medium. The cells were cultured under four distinct conditions (three inserts per condition per animal): (a) one-step approach under 5% O_2_; (b) one-step approach under 18% O_2_; (c) two-step approach under 5% O_2_; (d) two-step approach under 18% O_2_. The cultures were maintained parallel in two incubators (Heracell 150i/Steri-Cycle i160, ThermoFisher Scientific, USA) set at 37 °C, 5% CO_2_, and either 5% or 18% O_2_. Prior to the medium change, all media were pre-equilibrated in their respective incubators for 1 h, and the media were refreshed twice weekly.

#### One-step approach

**Fig. 1:**
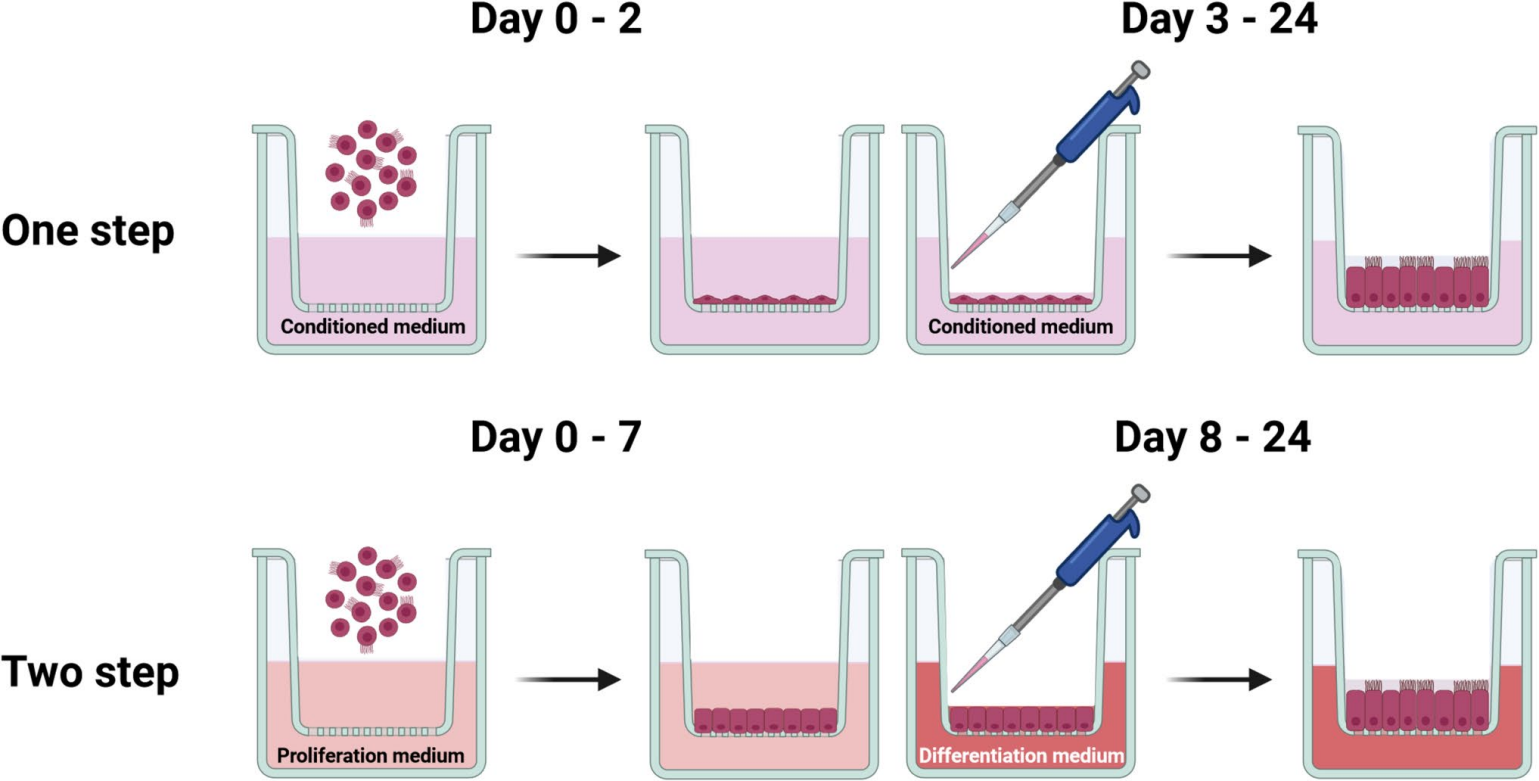
Schematic diagram illustrating the one-step and two-step approaches for the ALI-POEC culture procedures. *ALI* air–liquid interface, *POEC* porcine oviduct epithelial cells. Figure created with BioRender.com

The one-step approach is based on a conditioned medium comprising nutrients and growth factors produced by a mouse embryonic fibroblasts cell line (NIH/3T3, ATCC CRL-1658) to support the growth and differentiation of oviduct epithelial cells throughout the entire culture period, as previously reported by our group (Miessen et al. 2011; Chen and Schoen 2021). Briefly, a stock of 3T3-enriched medium was initially generated using the NIH/3T3 cell line (Miessen et al. 2011). The final conditioned medium was prepared with two parts of Ham’s F-12 supplemented with 10% fetal bovine serum (FBS) and one part of the 3T3-enriched medium. It was then supplied with 0.05 mg/ml gentamycin, 1 μg/ml amphotericin B, 100 U/ml penicillin, 100 μg/ml streptomycin, 0.01 mg/ml ascorbic acid (A4544, Sigma-Aldrich, USA) and 0.01 mg/ml glutathione (G6013, Sigma-Aldrich, USA). Cells were maintained in the conditioned medium at the liquid–liquid interface for 2 days and subsequently differentiated at the ALI until day 24, as illustrated in Fig. 1.

#### Two-step approach

The two-step approach employed a proliferation medium for the liquid–liquid interface stage until day 7, and a differentiation medium for the ALI stage until day 24, as previously described (Chen et al. 2017; Chen and Schoen 2021) (Fig. 1). Both media were formulated on the basis of a basic medium comprising DMEM/Ham’s F-12 with 2.5 mM L-glutamine, 100 U/ml penicillin, 100 μg/ml streptomycin, 0.25 μg/ml amphotericin B and 15 mM HEPES. The proliferation medium was the basic medium supplemented with 10 µg/ml insulin (I6634, Sigma-Aldrich, USA), 5 µg/ml transferrin (T8158, Sigma-Aldrich, USA), 25 ng/ml epidermal growth factor (E4127, Sigma-Aldrich, USA), 0.1 μg/ml cholera toxin (C8052, Sigma-Aldrich, USA), 30 µg/ml bovine pituitary extract (P1476, Sigma-Aldrich, USA), 5% FBS and freshly added 0.05 μM retinoic acid (R2625, Sigma-Aldrich, USA). The differentiation medium was prepared by supplementing the basic medium with 3% FBS, 2% Nu-Serum growth medium supplement (355100, Corning, USA) and 0.05 μM retinoic acid.

### Histology and histomorphometry

Bouin’s solution was freshly prepared just prior to use by mixing picric acid solution (P6744, Sigma-Aldrich, USA), 35% formaldehyde (4980.1, Carl Roth, Germany) and glacial acetic acid (45726, Sigma-Aldrich, USA) in a volume ratio of 15:5:1. For fixation of the obtained oviduct tissues, they were immersed in Bouin’s solution and kept in the refrigerator overnight. In the case of ALI-POEC cultures, on day 24, one insert per animal per condition was randomly selected for histological fixation immediately after measuring TEER (see next section), following a procedure previously described by our group (Chen and Schoen 2021). In brief, the membranes were gently washed with warm DPBS, and then the apical and basal compartments were filled with 0.2 ml and 1 ml of Bouin’s solution, respectively, for a 2-h incubation period. Subsequently, the membranes were carefully excised and vertically embedded in 1.5–2% agarose (16500-500, Invitrogen, USA), followed by post-fixation in 4% formaldehyde (P087.3, Carl Roth, Germany) for 1 h.

Following fixation, the oviduct tissues and POEC cultures were dehydrated using a series of ascending graded ethanol solutions (80–99%), cleared with Shandon xylene substitute (10128638, ThermoFisher Scientific USA) and then embedded in Paraplast Plus (39602004, Leica Biosystems, Germany). Afterwards, five 3-μm sections per sample were crafted for hematoxylin–eosin (HE) staining and analysed using a Zeiss Axio Imager A1 microscope (Carl Zeiss, Germany), coupled with the AxioVision SE64 Rel. 4.9 image analysis software. For the purpose of histomorphometry, five images were captured at ×400 magnification for each individual section. These images were subsequently subjected to analysis using ImageJ software (Bethesda, USA) to assess cellular features, including total cell counts, epithelial height and secretory cell numbers. The differentiation status of POEC cultures was evaluated using a set of morphological criteria encompassing ciliation, polarity, confluency, uniformity and monolayer formation, as outlined in Table 2. The maximum achievable score for a fully differentiated epithelial structure was 8 points.

**Table 2:** Morphological scoring system for the ALI-POEC cultures. Maximum score for a fully differentiated culture is 8 points

Score	Ciliation	Polarity	Confluency	Uniformity	Monolayer
2	Moderate/dense cilia	≥ 10 μm	Fully confluent	–	–
1	Few cilia	5–10 μm	Partly confluent	Homogeneous	Monolayer
0	No cilia	≤ 5 μm	Not confluent	Inhomogeneous	Partly multilayer

### Transepithelial electrical resistance (TEER) measurement

To test epithelial barrier formation, TEER measurement was performed before harvesting in all intact inserts using an EVOM2 Epithelial Voltohmmeter (WPI, USA) as detailed in our previously published book chapter (Chen and Schoen 2021). Inserts that displayed evident mechanical damage to the epithelial layer or ruptured membrane resulting from pipette or electrode handling were excluded. The average TEER value from three replicates of inserts was adopted to represent the value for each animal under a specific experimental condition. The new inserts, which contained only the corresponding culture medium during the one-step and two-step procedures without cells, were designated as blank controls. The readings from the corresponding controls were subsequently subtracted from the sample readings to determine the actual resistance of the samples. To calculate the unit area resistance (Ω∗cm^2^), the true resistance of the sample was multiplied by the membrane area (0.3 cm^2^ for a 24-well insert).

### Oviduct fluid surrogates (OFS) collection and protein quantification

On day 21, the accumulated OFS on the apical side was aspirated, followed by a careful rinse of the apical side with pre-equilibrated DMEM/Ham’s F-12. After a 72-h interval, the regenerated OFS in the apical compartment was collected from each insert, avoiding contact with the cellular layer. The collected OFS was subjected to two rounds of centrifugation at 2000 × *g* for 20 min at 4 °C, to eliminate any cellular debris. The resultant OFS was aliquoted and stored at − 70 °C until further use.

Quantification of the protein concentration within the OFS was performed in duplicate using the Micro BCA™ Protein Assay Kit (23235, ThermoFisher Scientific, USA), adhering to the manufacturer’s instructions. The absorbance of the samples was measured at 562 nm using a microplate reader (Infinite 200, TACAN, Switzerland) after a 2-h incubation at 37 °C.

### Western blot

Western blot analysis was performed to assess the presence of oviduct-specific glycoprotein (OVGP1) in the OFS. For each sample, an aliquot of 5 μl OFS was mixed with Pierce™ Lane Marker reducing sample buffer (39000, ThermoFisher Scientific, USA) according to the manufacturer’s instruction and boiled at 95 °C for 5 min. Oviduct epithelial cells isolated from one animal were lysed with RIPA buffer and served as a reference sample for both gels (10 μg/lane). The denatured proteins were separated by electrophoresis in 8% SDS–polyacrylamide gels and then electro-transferred onto polyvinylidene difluoride (PVDF) membranes (IPVHC0010, Merck Millipore, USA). Afterwards, the membranes were blocked with 5% non-fat dry milk (T145.2, Carl Roth, Germany) dissolved in PBS/0.2% Tween 20 (9127.1, Carl Roth, Germany) for 1 h at room temperature. Following the blocking step, membranes were incubated with rabbit anti-human OVGP1 primary antibody (ab118590, Abcam, UK, 1:1500, RRID:AB_10898500; the manufacturer validated the anti-OVGP1 primary antibody using human fallopian tube) diluted in PBS/0.2% Tween 20 containing 5% bovine serum albumin (8076.2, Carl Roth, Germany) overnight at 4 °C. The membranes underwent three rounds of washing, each lasting 10 min, using PBS/0.2% Tween 20. Subsequently, the membranes were subjected to incubation for 1.5 h at room temperature with HRP-conjugated goat anti-rabbit IgG antibody (7074S, Cell Signaling Technology, USA, 1:2000, RRID:AB_2099233). Chemiluminescence detection was carried out using the ECL^TM^ Prime Western Blotting Detection Reagent (RPPN2232, GE Healthcare, USA). Blot images were visualized by the AzureSpot system (Azure Biosystems, USA) and later analysed by the AzureSpot analysis software ‘1D gel/Western Blot Analysis’ for band quantification. The area of interest comprising the main OVGP1 signal was manually identified on the image, and lanes were automatically defined using the lane creation mode. The background intensity was subtracted from each blot using the lane edge subtraction method. To maintain consistency, same-sized areas for the major OVGP1 band in each lane were selected to assess the signal intensity. Afterwards, the signal intensity of OVGP1 in each sample lane was normalized against the density of the main OVGP1 band in the reference sample (oviduct cells) on the same gel image. To assess the abundance of OVGP1 in the complete OFS, the normalized signal intensity per microlitre was multiplied by the total volume of OFS produced by the corresponding sample.

### RT-qPCR analysis

For gene expression analysis in ALI-POEC, subsequent to TEER measurement, total RNA was isolated from two inserts per animal per culture condition using the NucleoSpin RNA kit (740955.50, Macherey–Nagel, Germany). RNA quantity and quality were assessed using the NanoDrop™ 2000c (ThermoFisher Scientific, USA) and Agilent 2100 Bioanalyzer (Agilent Technologies, Germany), respectively. To prepare cDNA, 1 µg of total RNA was reverse transcribed using the RevertAid reverse transcriptase (EP0441, ThermoFisher Scientific, USA), following the procedure previously described (Palma-Vera et al. 2017). Similarly, qPCR was conducted in duplicate utilizing the SensiFast™ SYBR No-ROX reagents (BIO-98020, Bioline Reagent, USA) in a LightCycler 96 (Roche, Germany) as previously documented (Palma-Vera et al. 2017). The PCR program involved an initial step at 95 °C for 10 min, followed by 40 cycles of 95 °C for 5 s, 60 °C or 62 °C for 15 s, 72 °C for 10 s, and a final melting step at 65 °C for 5 s, and 97 °C for 1 s.

To assess primer efficiency, a standard curve was generated for all primers by conducting a series of 1:10 dilutions of PCR products, ensuring an efficiency ranging between 90% and 100%. Adjusted for the individual primer efficiency, 2^−∆∆CT^ methodology was used to calculate the relative gene expression (Livak and Schmittgen 2001). The geNorm algorithm was used to determine the stability of four housekeeping genes, including actin beta (*ACTB*), succinate dehydrogenase complex flavoprotein subunit A (*SDHA*), glyceraldehyde-3-phosphate dehydrogenase (*GAPDH*), and transforming growth factor β-stimulated clone 22 domain family member 2 (*TSC22D2*) (Perkins et al. 2012). A normalization factor was calculated on the basis of the geometric mean of the most stable reference genes (*ACTB* and *SDHA*). Detailed information about the primers is listed in Table 3.

**Table 3:** Primer sequences for RT-qPCR with annealing temperatures

Gene	Forward primer (5′ to 3′)	Reverse primer (5′ to 3′)	Annealing temperature (°C)	Length (bp)
*ACTB*	CAACTGGGACGACATGGAG	GAGTCCATCACGATGCCAG	60	234
*AQP1*	TCATCAGCATCGGTTCAGCCCT	GAGTTGTCGGGCAGAGAGGAGG	62	298
*AQP3*	TTGTATTACGATGCGATCTGGG	AGAGTTGAAGCCCATTGAGG	62	267
*ATP1A1*	TCATCCCATCACAGCCAAAG	GTCTTCGACCGTTTCATTGC	60	73
*ESR1*	AGGGAAGCTCCTGTTTGCTCC	CGGTGGATATGGTCCTTCTCT	60	234
*GAPDH*	ATTCCACCCACGGCAAGTTC	AAGGGGCAGAGATGATGACC	60	225
*MKI67*	TCGTAAGTGCTTCTGTGTCTG	CTGTCCTCTGCTCATCCATTAC	60	139
*MUC16*	AGTGGCTATGCACCCCAGAC	ACCAGGCAGGAGCGGAATAC	60	191
*OVGP1*	GGGGCACTTTCTGTGGCACT	AGCCAGGCTTTCAGGGCAAG	60	149
*PGR*	TGAGAGCACTAGATGCCGTTGCT	AGAACTCGAAGTGTCGGGTTTGGT	60	197
*SCNN1A*	ACCGCTTCCACTACATCAAC	CGAAGATGAATTTGCCCAGTG	60	87
*SDHA*	CTACAAGGGGCAGGTTCTGA	AAGACAACGAGGTCCAGGAG	60	141
*TSC22D2*	AGACTCAGACCCAGCCTTTG	CAACAGGAGGCTTCACAACA	60	120

### Statistical analysis

**Fig. 2:**
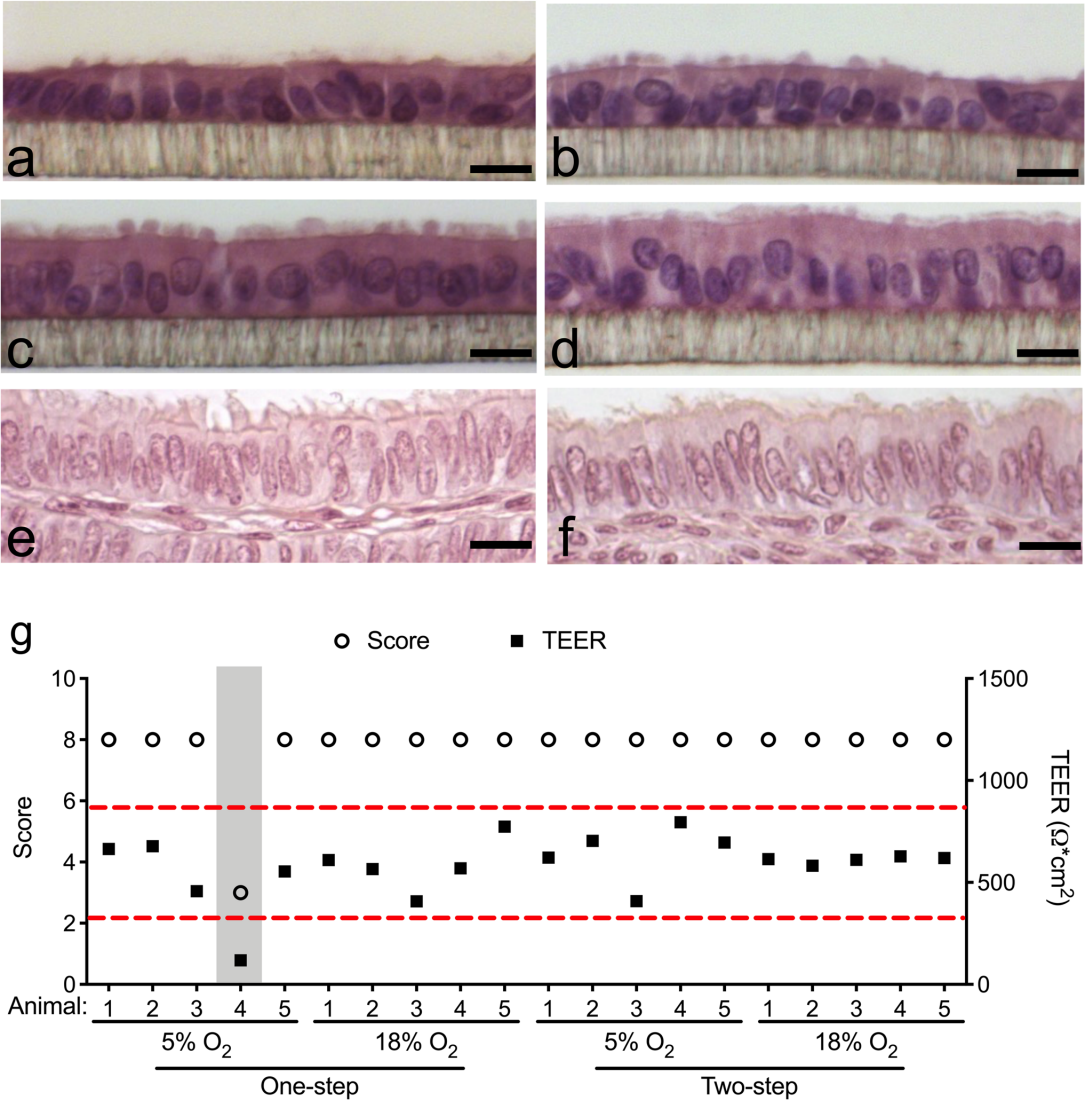
ALI-POEC exhibited in vivo-like morphology and epithelial barrier formation under all tested cell culture conditions. **a**–**f** POEC morphology in vitro and in vivo (HE staining, magnification ×400, scale bar = 20 µm). ALI-POEC cultivated under 5% O_2_ in one-step (**a**), 18% O_2_ in one-step (**b**), 5% O_2_ in two-step (**c**) and 18% O_2_ in two-step approach (**d**); tissue section of porcine oviduct ampulla (**e**) and isthmus (**f**) originating from a non-cycling 6-month-old gilt. **g** Morphology scores of ALI-POEC cultures and corresponding TEER values. Left y-axis, morphological scores; right y-axis, TEER values (Ω∗cm^2^). Red dashed lines indicate the TEER range for morphologically intact and differentiated ALI cultures. Shaded area specifically shows animal 4 under 5% O_2__one-step media regime, which exhibited poor differentiation and low TEER, and was therefore excluded from further analyses. *ALI* air–liquid interface, *POEC* porcine oviduct epithelial cells, *TEER* transepithelial electrical resistance

Because one sample set (animal 4 under one-step approach with 5% O_2_) demonstrated notably low TEER (Fig. 2g) that fell outside the suitable range for differentiated ALI-POEC culture (Chen et al. 2015), this particular sample set was excluded from subsequent analysis. Data were all analysed using the IBM SPSS Statistics 24 software (USA). The normality of datasets was assessed by the Shapiro–Wilk test. For normally distributed data (*p* > 0.05) as well as *MUC16*, in which the variance is homogeneous (*p* = 0.32) and data is approximately normal (*p *values within subgroups are 0.19, 0.15, 0.79, 0.05), the two-way analysis of variance (ANOVA) was applied, followed by Student’s *t* test (Larson 2008; Krzywinski and Altman 2014). For data (*PGR, AQP3*) that did not conform to a normal distribution, the non-parametric Kruskal–Wallis test was carried out followed by the Mann–Whitney *U* test. We consider statistical significance if *p* < 0.05.

## Results

### Impact of O_2_ levels and media regimes on ALI-POEC structure and composition

**Fig. 3:**
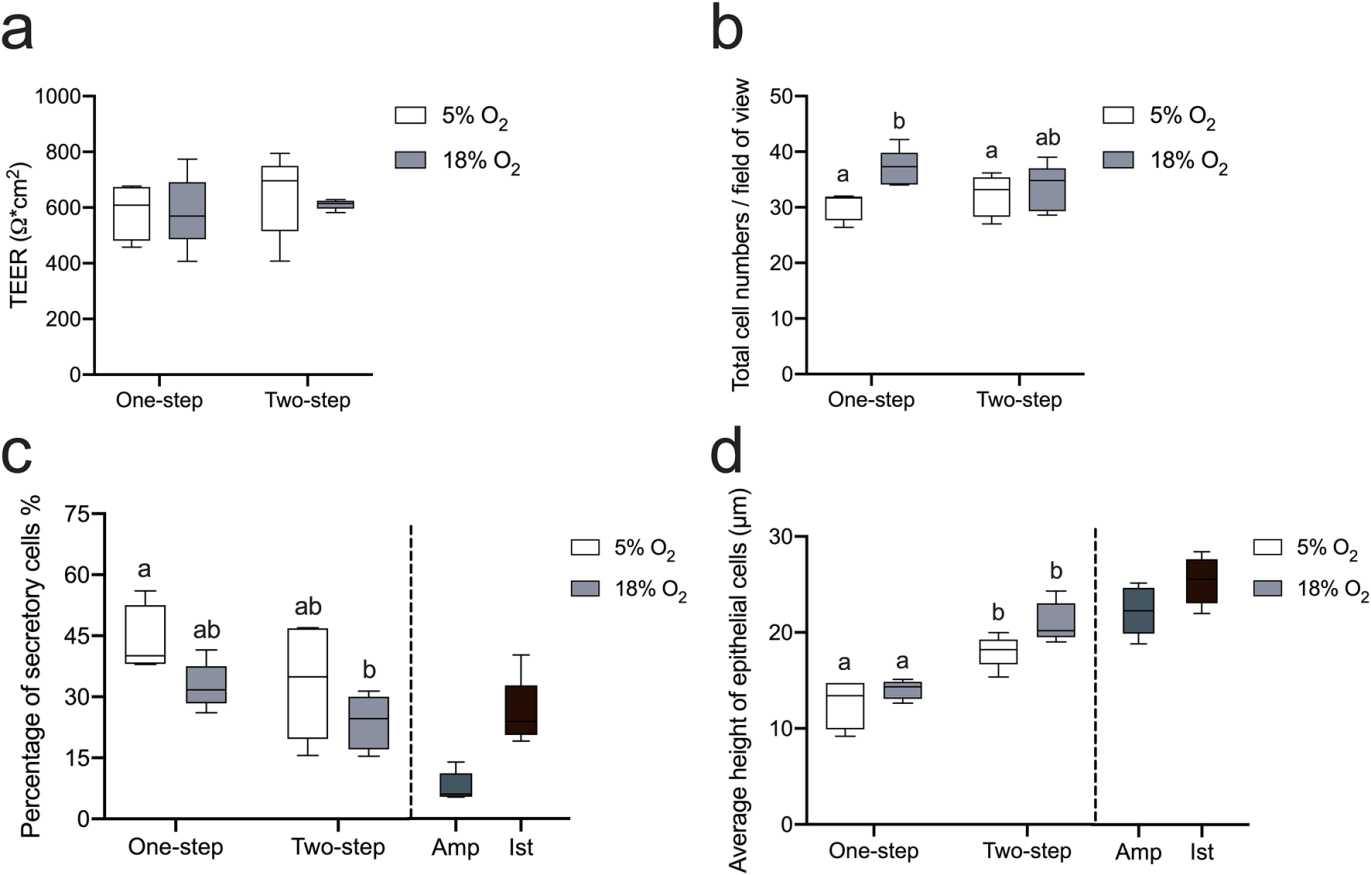
Morphological assessment and barrier formation of ALI-POEC in response to diverse O_2_ levels and media regimes. **a** TEER of POEC after 24 days cultivation. **b** Cell counts per field of view following HE staining. **c** Ratio of secretory cells to the total cell count in ALI-POEC and oviduct tissues. **d** Average height of epithelial cells in ALI-POEC and oviduct tissues. Different superscript letters indicate statistical significance (*p* < 0.05) among subgroups. *N* = 5 animals/culture condition (except for the 5% O_2__one-step condition, involving 4 animals). *ALI* air–liquid interface, *POEC* porcine oviduct epithelial cells, *TEER* transepithelial electrical resistance. *Amp* oviductal ampulla region collected from 5 animals, *Ist* oviductal isthmus region collected from 5 animals

After 3 weeks of cultivation at the ALI, regardless of the O_2_ levels and media regimes, cultures under all conditions typified the monolayered oviduct epithelium, featuring a distinct arrangement of columnar-shaped ciliated and secretory cells (Fig. 2). Morphological criteria, including ciliation, polarity, confluency, uniformity and the formation of a monolayer, were employed to further evaluate the culture quality. Almost all cultures achieved the highest quality score of 8, reflecting their well-differentiated status, except for a single culture from animal 4 (A4_5% O_2__one-step). In cultures that scored the maximum 8, tight junction development was confirmed by moderate TEER values within the range of 408.30–794.95 Ω∗cm^2^ (Figs. 2g and  3a). Contrarily, culture A4_5% O_2__one-step exhibited low electrical resistance of 118.35 Ω∗cm^2^. This specific culture displayed a less uniform structure, characterized by flat and squamous-like cells. Notably, considering the recommended TEER range for good quality ALI cultures from the porcine oviduct, as proposed in a prior paper (Chen et al. 2015), this particular culture was excluded from subsequent analysis.

The histomorphometry analysis unveiled a significant influence of the O_2_ level employed during culture on total cell numbers/field of view (*p* < 0.05, Fig. 3b). Both O_2_ tensions and media regimes affect the cell populations (*p* < 0.05, Fig. 3c). Both O_2_ levels (*p* < 0.05) and media regimes (*p* < 0.01) strongly impact cell polarization (Fig. 3d). Cultures maintained in the two-step approach displayed greater cellular height, averaging 18.02 ± 2.39 µm, which is comparable to what is observed in tissue.

### O_2_ levels govern the OFS production

**Fig. 4:**
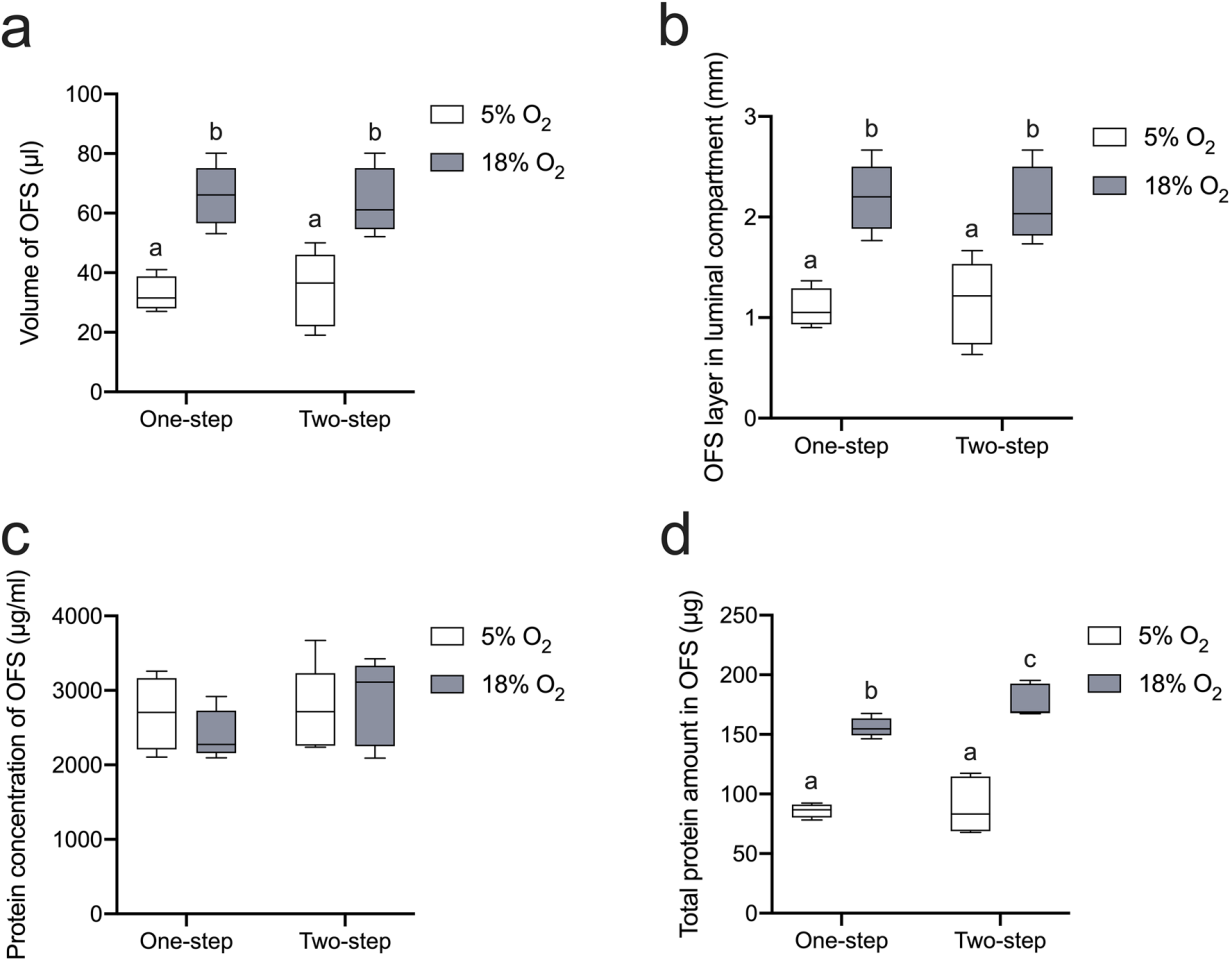
Characterization of OFS derived from ALI-POEC in response to various O_2_ conditions and media regimes. **a** Volume of OFS generated in the apical compartment of 24-well inserts over a period of 3 days. **b** Average thickness of OFS layer in the apical compartment. **c** Protein concentration of OFS quantified by Micro BCA. **d** Total protein amount in complete OFS. *N* = 5 animals/culture condition (except for the 5% O_2__one-step condition, involving 4 animals). Significant differences (*p* < 0.05) are indicated between subgroups with different superscript letters. *OFS* oviduct fluid surrogates, *ALI* air–liquid interface, *POEC* porcine oviduct epithelial cells

Once differentiated, ALI-POEC constantly produced a layer of OFS in the apical compartment. The freshly generated OFS was collected over a 3-day period, and its volume was measured. It is worth noting that all the collected OFS samples are transparent in colour, and clearly distinguishable from the phenol red-containing medium in the basal compartment. The O_2_ levels exert a significant influence on the volume of OFS and, therefore, the thickness of OFS in the insert (*p* < 0.01, Fig. 4a, b). Specifically, the volume of OFS was substantially increased under 18% O_2_, averaging 64.9 ± 10.10 µl, in contrast to the 5% O_2_ condition, where it averaged 33.72 ± 9.63 µl. Consequently, the calculated thickness of the OFS layer within 24-well insert increased from 1.12 ± 0.32 mm under 5% O_2_ to 2.16 ± 0.34 mm under 18% O_2_ (Fig. 4b). Interestingly, the protein concentration of OFS remained consistent across different conditions (Fig. 4c). Altogether, this led to a markedly elevated total protein content within the OFS under 18% O_2_ as opposed to 5% O_2_ (*p* < 0.01, Fig. 4d).

### O_2_ levels and media regimes influence the presence of OVGP1 protein in OFS

**Fig. 5:**
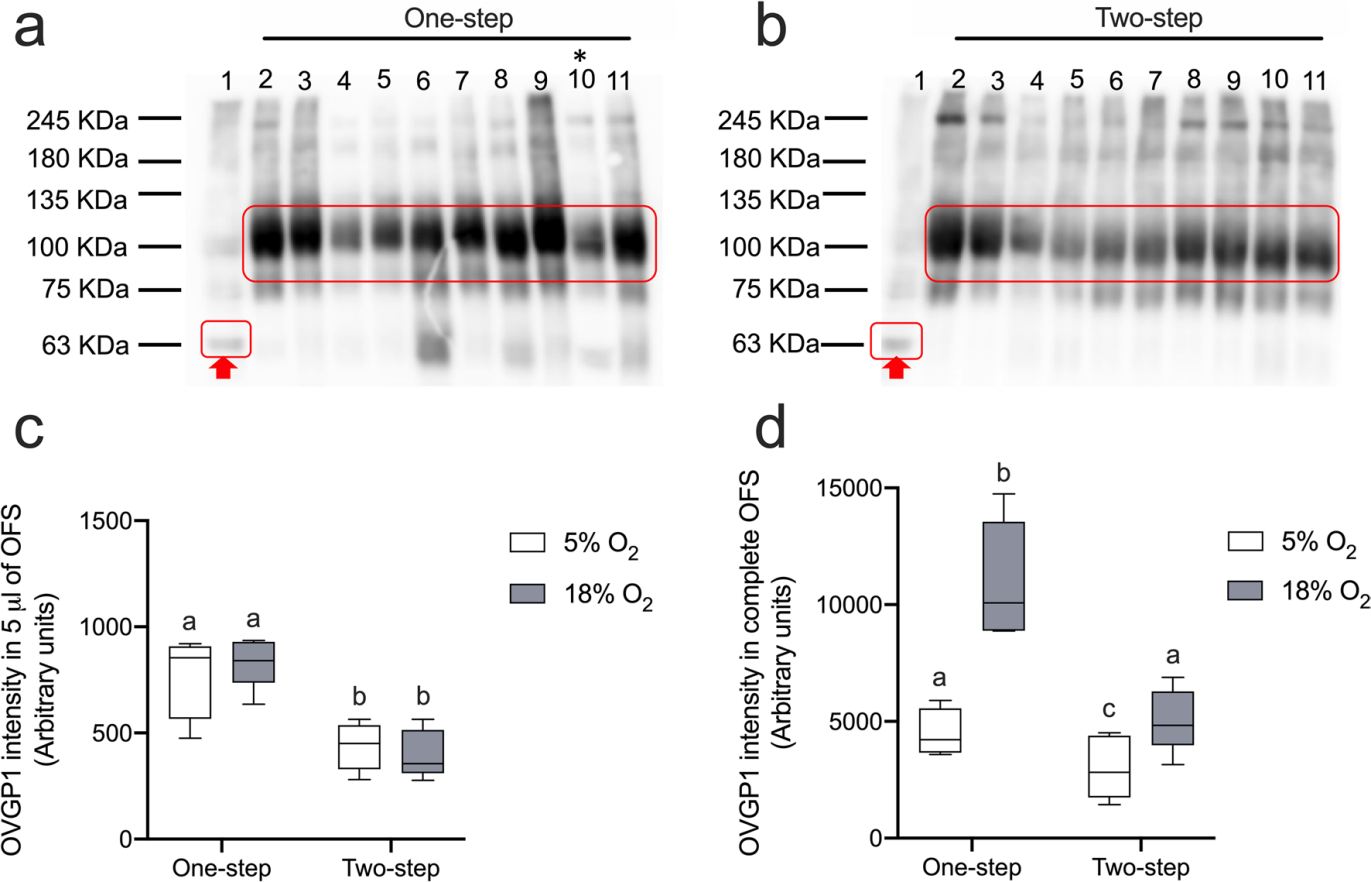
Western blot and immunodetection of OVGP1 in OFS derived from ALI-POEC under different culture conditions. **a** One-step media regime. **b** Two-step media regime. On both gel images, lane 1 represents 10 μg of oviduct epithelial cell lysate, serving as reference sample; lanes 2, 4, 6, 8 and 10 (5% O_2_ condition) represent 5 µl of OFS samples derived from A1, A2, A3, A5 and A4, respectively; lanes 3, 5, 7, 9 and 11 (18% O_2_ condition) represent 5 µl of OFS samples derived from A1, A2, A3, A5 and A4, respectively. **c** The chemiluminescence intensity after OVGP1 immunodetection in 5 µl of OFS. **d** Calculated OVGP1 abundance in the complete OFS volume produced over 3 days. The chemiluminescence signal intensities of the main OVGP1 band in each lane (indicated by the red rectangular) were normalized to the main band of the reference sample (red arrow) on the same membrane. Significant differences (*p* < 0.05) are marked between subgroups with different superscript letters. *OFS* oviduct fluid surrogates, *ALI* air–liquid interface, *POEC* porcine oviduct epithelial cells, * indicates OVGP1 expression from animal 4 under 5% O_2__one-step media regime, which was excluded from the analysis

To further evaluate the secretion of OVGP1 by ALI-POEC into the apical pole under varying O_2_ and medium conditions, immunoblot was performed using 5 µl of OFS collected from each sample. The results confirmed the abundance of OVGP1 in the OFS across all culture conditions (Fig. 5a, b). The signal intensity analysis revealed a significant disparity in OVGP1 protein concentration between the one-step and two-step media regimes, with the former exhibiting notably higher levels (*p* < 0.01, Fig. 5c). When considering the overall abundance of OVGP1 within the entire OFS, it was significantly affected by O_2_ tension (*p* < 0.01), medium choice (*p* < 0.01) and interaction between O_2_ level and media regime (*p* < 0.05, Fig. 5d). To sum up, a higher total amount of OVGP1 was yielded in the OFS under the one-step regime compared to the two-step regime in the same O_2_ environment; additionally, 18% O_2_ condition boosted the total OVGP1 amount in contrast to 5% O_2_, within both media regimes (Fig. 5d).

### O_2_ levels and media regimes affect gene expression related to ion channels

**Fig. 6:**
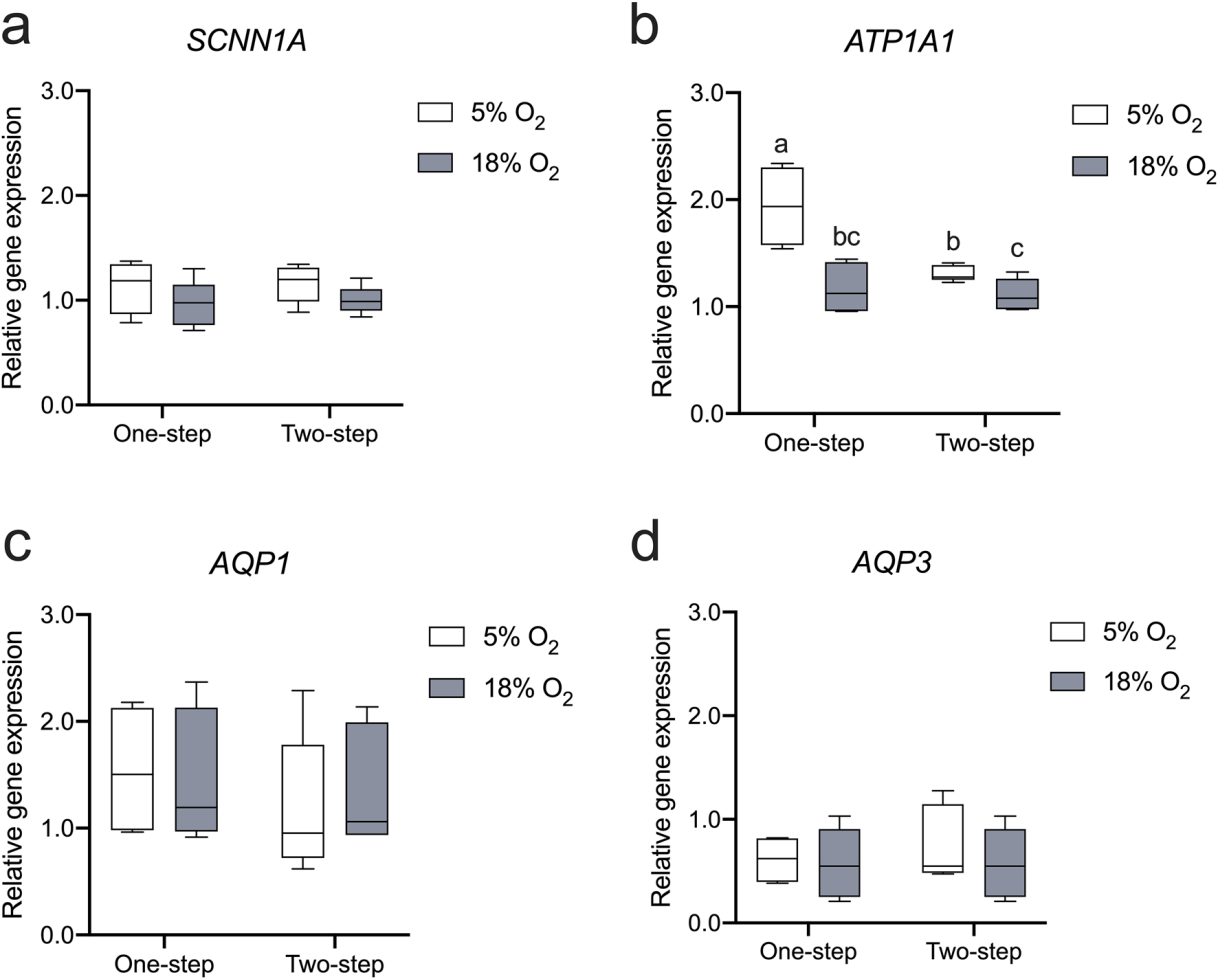
Expression of genes related to ion channels and water transporters in response to diverse O_2_ levels and media regimes. Relative mRNA expression of *SCNN1A* (**a**), *ATP1A1* (**b**), *AQP1* (**c**) and *AQP3* (**d**) in ALI-POEC. *N* = 5 animals/culture condition (except for the 5% O_2__one-step condition, involving 4 animals). Different superscript letters indicate statistical significance (*p* < 0.05) between subgroups. *ALI* air–liquid interface, *POEC* porcine oviduct epithelial cells

Given the significant variations in the fluid volume of OFS triggered by O_2_ levels (Fig. 4a), we conducted further analysis on the expression of genes related to transepithelial fluid transport, including water transporters and ion channels that actively transport salt across epithelium, thereby driving the flow of water. The epithelial sodium channels (ENaC) are apically located in the oviduct and function to transport sodium ions (Na^+^) from the luminal fluid into the cytoplasm. In our study, we did not observe any significant alterations in the expression of *SCNN1A*, a gene responsible for encoding the alpha subunit of the epithelial sodium channel (ENaC) (Fig. 6a). The Na^+^/K^+^-ATPase locates in the basolateral membrane of epithelium, which pumps Na^+^ out of cytoplasm into the interstitial side. The expression of the *ATP1A1* gene, encoding the subunit alpha 1 of Na^+^/K^+^-ATPase, showed a significant response to the different O_2_ levels (*p* < 0.01), media regimes (*p* < 0.01) and the interaction between O_2_ and medium conditions (*p* < 0.05, Fig. 6b). Specifically, under the same media regime, the expression of *ATP1A1* was significantly downregulated by 18% O_2_ compared to 5% O_2_ (Fig. 6b). Additionally, we assessed the expression of aquaporins (*AQPs*) which offer the transepithelial route for water transport in the direction of osmotic gradients. The classical aquaporin (*AQP1*) and aquaglyceroporin (*AQP3*) were both present in the ALI-POEC culture but did not exhibit any significant response to either the O_2_ tensions or media regimes (Fig. 6c, d).

### O_2_ levels and media regimes differentially modulate oviductal gene expression

**Fig. 7:**
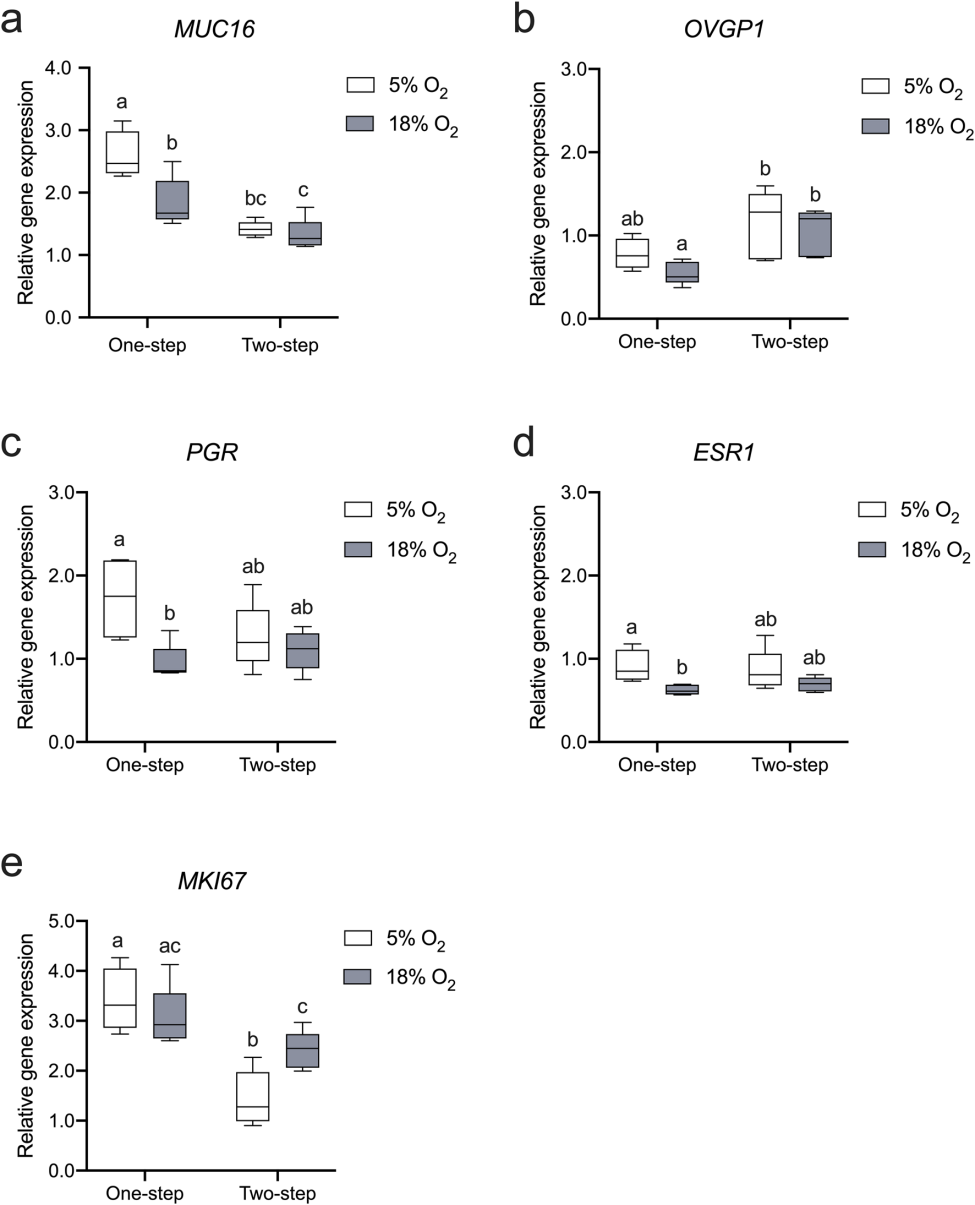
Expression of genes related to oviduct functionality, hormone signalling and cell proliferation in response to various O_2_ levels and media regimes. Relative mRNA abundance of *MUC16* (**a**), *OVGP1* (**b**), *PGR* (**c**), *ESR1* (**d**) and *MKI67* (**e**) in ALI-POEC. *N* = 5 animals/culture condition (except for the 5% O_2__one-step condition, involving 4 animals). Statistical significances are indicated (*p* < 0.05) between subgroups with different superscript letters. *ALI* air–liquid interface, *POEC* porcine oviduct epithelial cells

The expression of functional genes in the oviduct, such as mucins and steroid hormone receptors, was assessed using qPCR. Notably, the transcription of mucin 16 (*MUC16*), which encodes a mucus glycoprotein found on the apical surface of the oviduct epithelium, exhibited a significant response to different O_2_ levels (*p* < 0.01), media regimes (*p* < 0.01) and the interaction between O_2_ and medium conditions (*p* < 0.05, Fig. 7a). Specifically, the mRNA abundance of *MUC16* was observed to be approximately two times higher under the one-step regime compared to the two-step regime (Fig. 7a). Moreover, within the one-step regime, the expression of *MUC16* was significantly lower under 18% O_2_ compared to 5% O_2_ (Fig. 7a). Significant differences were noted in the context of media regimes concerning the expression of *OVGP1* (*p* < 0.01, Fig. 7b). Specifically, under an 18% O_2_ environment, *OVGP1* mRNA production exhibited higher levels within the two-step regime compared to the one-step regime. Concerning *PGR*, under the conditions of 5% O_2_ as opposed to 18% O_2_ within the one-step regime, there was a distinct increase in its transcription (Fig. 7c). The expression of estrogen receptor 1 (*ESR1*) was also significantly influenced by O_2_ levels (*p* < 0.05, Fig. 7d). Remarkably, expression of *ESR1* exhibited an identical pattern to that of *PGR* (Fig. 7d). We also assessed the expression of the proliferation marker Ki-67 (*MKI67*), revealing a substantial contrast between the one-step and two-step regimes (*p* < 0.01, Fig. 7e). Within the two-step regime, 18% O_2_ led to a higher *MKI67* expression compared to the 5% O_2_ condition.

## Discussion

The oviduct epithelium is a polarized monolayer, with the apical pole oriented towards the oviduct lumen, where critical early reproductive events occur in a reduced O_2_ environment (Coy et al. 2012). In recent decades, our group and others have embraced the ALI culture approach, using one-step or two-step media regimes as outlined in Table 1, for in vitro cultivation of oviduct epithelial cells from diverse mammalian species. The culture conditions were specified in 26 out of the 28 studies listed. Among these, 23 were conducted under atmospheric conditions with 5% CO_2_ in a humidified incubator, resulting in supraphysiological O_2_ levels of around 18%. Exceptions include the study by Ferraz et al., which performed bovine oviduct and embryo co-culture on a chip under 7% O_2_, and our group’s investigations into the influence of elevated cortisol due to maternal stress on the oviduct microenvironment under 5% O_2_ conditions (Ferraz et al. 2018; Du et al. 2020, 2022).

In conventional 2D culture methods, cells are typically submerged under several millimetres of culture medium (Place et al. 2017; Tse et al. 2021), and O_2_ must diffuse through this medium to reach the cells, resulting in significantly reduced O_2_ levels in the immediate pericellular environment (Stuart et al. 2018). By contrast, under the ALI condition, the O_2_ availability is improved (Nossol et al. 2011): although no medium is applied to the apical side, the apical cell surface remains hydrated and is directly exposed to the gas conditions within the incubator. As the headspace gas settings are approximately what the cells experience, it emphasizes the necessity to maintain physiological oxygenation in ALI cultures. An alternative path for O_2_ diffusion occurs within the culture medium in the basal compartment, where it traverses the filter membrane before entering cells via the basolateral plasma membrane. In addition to the diffusion distance through the medium, the filter membrane can serve as a significant physical barrier in this process. Depending on the filter membrane’s pore size and pore density, only approximately 0.5–12.6% of its surface area is porous and permeable (calculated on the basis of product specifications). Consequently, the contribution of O_2_ from the basal path is much less significant.

Previous research has shown that O_2_ levels have a profound impact on the proliferation and differentiation of 2D cultured cells. The effects vary depending on the specific cell types, with high O_2_ levels capable of either promoting or inhibiting the differentiation process (Alva et al. 2022a). In compartmentalized cultures employing filter membranes, elevated O_2_ levels have consistently been demonstrated to improve the differentiation of epithelial cells derived from the intestine, oviduct and airway (Nossol et al. 2011; Miessen et al. 2011; Gerovac et al. 2014; Kouthouridis et al. 2021). In our study, we observed that when compared to a 5% O_2_ environment, a higher O_2_ level of 18% accelerated the differentiation of oviduct epithelial cells. This acceleration was evident through notable increases in epithelial height, proportion of ciliated cells, and reduced lateral dimensions of cells. Our findings closely align with a recent study involving human bronchial epithelial cells, where exposure to hyperoxic conditions (30% O_2_) similarly promoted several changes, including increased epithelial layer thickness, larger ciliated cell areas and a shift towards a laterally more compact morphology (Kouthouridis et al. 2021). Gerovac et al. also reported similar results in their research, revealing that reduced O_2_ levels suppressed cilia formation and the expression of key genes related to ciliogenesis in human bronchial epithelial cells maintained at the ALI (Gerovac et al. 2014). They suggested that O_2_ availability plays a role in mediating the differentiation of epithelial cells, possibly through the Notch signalling pathway. The findings based on the differentiation-supporting ALI system emphasize that elevating O_2_ availability, although beyond physiological norms, moves epithelial cells toward the terminal differentiation process.

In our previous study, we characterized the apical fluid generated by ALI-POEC and demonstrated its capability to represent oviduct fluid in supporting embryo development (Chen et al. 2017). It is striking to observe that the O_2_ levels in the apical compartment substantially influence the generation of OFS by ALI-POEC. Specifically, the volume of OFS exhibited a two-fold increase under 18% O_2_ compared to 5%. This substantial increase reflects alternations in the water transport routes by oviduct epithelial cells. Initially we checked the expression of epithelial sodium channels (ENaC) situated on the apical membrane. Since these channels are responsible for transporting Na^+^ from the apical fluid into the cytoplasm of epithelial cells, causing water to flow in the same direction (Alexander et al. 2019; Du et al. 2022). However, we did not detect a significant influence of O_2_ levels on the expression of *SCNN1A* (encoding the α subunit of ENaC). In contrast, the expression of *ATP1A1*, which encodes the subunit alpha 1 of Na^+^/K^+^-ATPase, was significantly downregulated in the presence of 18% O_2_. The Na^+^/K^+^-ATPase is basolaterally located and pumps Na^+^ ions out of the cells, leading to a net flow of Na^+^ towards the basolateral side. Exposure to 18% O_2_ decreased the expression of Na^+^/K^+^-ATPase, thereby resulting in a reduced net flow of Na^+^ towards the basal compartment. Consequently, the changes in osmotic potential may limit water movement towards the basolateral compartment (corresponding to the blood/interstitium side). This finding aligns with the observation that a greater volume of apical fluid accumulated under the 18% O_2_ environment. The media regimes also significantly affected the expression of *ATP1A1*, although the volume of OFS remained consistent across different media regimes. This suggests the involvement of other factors in the regulation of water transport through the epithelium, such as the water channel aquaporins (e.g. *AQP1*, *AQP3*, *AQP6* and *AQP9* in the oviduct) and cystic fibrosis transmembrane conductance regulator (*CFTR*) (Im et al. 2020). Further investigations are warranted to assess the activities of ion and water channels at the protein level to validate the mechanisms governing the OFS volume.

We propose that the increase in the volume of OFS serves as a protective mechanism against the high O_2_ level (18%) reaching the cell surface. Our results showed that the thickness of OFS increased from 0.63–1.67 mm under 5% O_2_ to 1.77–2.67 mm under 18% O_2_ inside the apical compartment of the insert. It has previously been shown in human bronchial epithelial cells that a 3-mm fluid layer on the 24-well filter insert is sufficient to create a lower O_2_ level at the epithelium surface while preserving the differentiated status, as observed by us.

The increase in volume of OFS in the presence of supraphysiological O_2_ (18%) is concomitant with heightened protein secretion activity into the apical side by epithelial cells, as evidenced by the fact that the protein concentration in OFS remained consistent under different O_2_ conditions. Overall, the 18% O_2_ environment induced a significantly greater protein abundance within the OFS. It is well established that OVGP1 is the major glycoprotein secreted into the oviduct fluid, contributing to various aspects, including oocyte maturation, sperm–oocyte binding and embryo development (Zhao et al. 2022). Our study identified high abundance of glycosylated forms of OVGP1 in the OFS. Although its concentration within the OFS was not affected by the O_2_ levels, supraphysiological O_2_ (18%) resulted in a higher total amount of secreted OVGP1. OFS production in response to environmental O_2_ is potentially important, given that oviduct fluid constitutes the microenvironment for a series of early reproduction events, particularly fertilization and early embryo development. Further research is necessary to examine the presence of other essential elements in OFS, such as electrolytes and metabolites.

It is well known that sex steroid hormones, particularly estrogen and progesterone, are the dominating hormones in the oviduct, controlling the epithelium structure and functionality via acting through nuclear and membrane receptors (Barton et al. 2020). We observed that exposure to the higher O_2_ level (18%) led to a significant downregulation in the expression of both *ESR1* and *PGR1*, which may initiate alterations in the downstream hormone signalling pathways within the oviduct epithelium. Our findings are consistent with previous studies that involved culturing various cell lines in parallel under either 5% or 18% O_2_, which demonstrated changes in the transcription of thousands of genes (Alva et al. 2022b).

In the past, our group has employed both the one-step and two-step media regimes to study the physiology of oviduct epithelial cells, consistently revealing gene expression changes that mimic in vivo responses towards steroid hormones (Chen et al. 2013a, 2018; Du et al. 2022). However, a direct comparison to assess the cell compositions and gene expression profiles of ALI-POEC under the two media regimes has yet to be undertaken. In our present work, histological analysis revealed varying epithelial thickness and cilia density of ALI-POEC maintained under different media regimes while exposed to the same O_2_ environment. Additionally, although the medium conditions did not change OFS volume and total protein abundance, the composition of proteins within OFS, as illustrated by OVGP1, varied significantly when subjected to different media regimes. The regulation of *MUC16* and *OVGP1* at the mRNA level provides evidence of the influence of the media on mucin production. Despite these differences, the physiological features and response to varying O_2_ levels remain broadly consistent under both media regimes, underscoring the suitability of both culture approaches in investigating oviduct epithelium physiology.

It is noteworthy that the levels of glucose, which serves as the primary energy source for cells, in the two media are distinct. The one-step media regime was based on Ham’s F12, containing 1802 mg/L of glucose, while the two-step media regime, based on DMEM/Ham’s F-12, consisted of 3151 mg/L of glucose. It has been suggested that glucose and O_2_ levels collectively determine the growth and metabolism of mouse myoblasts and human prostate cancer cells in response to resveratrol, and the impact differs between low and high glucose levels under identical O_2_ conditions (Fonseca et al. 2018). Therefore, the minor variations in response to O_2_ levels under different medium conditions may be attributed to differences in glucose availability.

In conclusion, to the best of our knowledge, our study provides the first evidence for functional differences in epithelium cells cultured under 5% O_2_ and 18% O_2_. While most oviduct cells typically encounter O_2_ levels ranging from 4% to 10% in vivo, standard cell cultures in a humidified incubator with 5% CO_2_ are maintained at approximately 18% O_2_ in vitro. The supraphysiological O_2_ (18%) level resulted in enhanced polarization and ciliogenesis of the epithelium, offering a potential strategy to pursue studies on the terminal differentiation of epithelial cells in vitro. However, this 18% O_2_ condition significantly affected the production of OFS, the crucial microenvironment for gametes and the early embryo, along with alterations in cellular constituents and expression of key oviduct functional genes. The oviduct epithelial cells adapted to the supraphysiological O_2_ environment, which may limit their capability to accurately represent the in vivo tissue. These results highlight the critical importance of creating a physiologically relevant gas environment for cell cultures in order to improve the reproducibility and translational relevance of in vitro studies.

## Data Availability

Data from this study will be available from the corresponding author upon reasonable request.
